# Gestational diabetes mellitus and its impact on the mother-infant relationship: A cohort study in the postnatal period

**DOI:** 10.1016/j.pmedr.2023.102270

**Published:** 2023-06-02

**Authors:** Madeleine Benton, Megan Davies, Khalida Ismail, Jacopo Lenzi

**Affiliations:** a Department of Psychological Medicine, King’s College London, United Kingdom; b Section of Epidemiology, University Copenhagen, Denmark; c Department of Biomedical and Neuromotor Sciences, University of Bologna, Italy

**Keywords:** Gestational diabetes mellitus, Mother–infant relationship, Perinatal mental health, Maternal health, Mother–infant bonding

## Abstract

Women with gestational diabetes mellitus (GDM) are at increased risk of poor perinatal mental health outcomes. However, the association between GDM and the mother–infant relationship is unclear. This study aimed to examine whether GDM itself impacts the mother–infant relationship and maternal mental health using a cohort study design. We used data from the Cohort of Newborns in Emilia-Romagna (CoNER) study, which included 642 women recruited in Bologna, Italy. Psychological data were collected at 6 and 15 months postnatally using a purpose designed measure to examine the mother–infant relationship. We used linear fixed effects and mixed-effects models to assess the effect of GDM on relationship scores at 6 and 15 months postpartum. Women with GDM had significantly lower relationship scores at 15 months postpartum [β − 1.75 95% CrI (−3.31; −0.21)] but not at 6 months [β − 0.27 95% CrI (−1.37; 0.81)]. Mother–infant relationship scores were significantly lower overall at 15 months compared to 6 months postpartum [β − 0.29 95% CrI (−0.56; −0.02)]. Our findings suggest that there may be a delayed effect on the mother–infant relationship in response to the experience of GDM. Future research using large birth cohorts should investigate this further to confirm these findings, and whether women with GDM would benefit from early interventions to improve relationships taking into account length of time postpartum.

## 1 Background

Gestational diabetes mellitus (GDM) is characterised by hyperglycaemia that first develops in pregnancy, and is associated with several adverse outcomes for both mother and infant, including obstetric complications (Zhu and Zhang, 2016). The global prevalence of GDM is approximately 15%, and prevalence rates are expected to continue rising (Wang et al., 2022). While GDM usually resolves after delivery, for the mother there is a 50% increased risk of a reccurance of GDM in subsequent pregnancies (Schwartz et al., 2015), and a nearly 10-fold higher risk of developing type 2 diabetes in later life (Vounzoulaki et al., 2020), whilst their child has an increased lifetime risk of obesity and type 2 diabetes (Deierlein et al., 2011, Farahvar et al., 2019). The intensive management of GDM, aiming for optimal blood glucose levels, is physically and psychologically demanding and has the potential to change the contextual experience of pregnancy from ‘normal’ to one that is highly medicalised (Parsons et al., 2018).

Mental health problems are now the commonest complication of the perinatal period (encapsulating the duration of pregnancy and the first year following birth), with one in five women experiencing a mental health problem during this time (Howard and Khalifeh, 2020, O’Hara and Wisner, 2014). Perinatal mental health problems are associated with adverse maternal, fetal, neonatal outcomes, and emotional and behavioural problems in the child (Stein et al., 2014). The relationship between GDM and depression may be bi-directional. There is some evidence that pre-pregnancy depressive symptoms heightens the risk of GDM (OuYang et al., 2021), and GDM increases the risk of perinatal mental health problems including depression. A recent systematic review showed that the pooled estimates of depression were increased by 2-fold in pregnant women with GDM compared to pregnant women without GDM (Wilson et al., 2020).

The mother-infant relationship is multifactorial and complex. It captures the connection between the mother and her baby involving cognitions and emotions towards the baby (Bicking Kinsey and Hupcey, 2013). The mother–infant relationship is important for mother and baby. An impaired mother–infant relationship can be a consequence of maternal depression (Śliwerski et al., 2020) and is associated with delayed child development, and subsequent development of psychopathology in adulthood (Moehler et al., 2006, Joas and Möhler, 2021). In addition to depression, other risk factors for an impaired relationship include negative feelings towards pregnancy, maternal stress, and anxiety (Moehler et al., 2006, Nakano et al., 2019, Bind et al., 2021, Lehnig et al., 2019, Rusanen et al., 2021). Importantly, these risk factors are also associated with the experience of GDM (Craig et al., 2020). Understanding whether GDM impacts the mother–infant relationship is necessary for developing appropriate interventions for women who may be at risk of impaired postpartum bonding and poor postpartum mental health. The clinical relevance is that the mother–infant relationship has been shown to be modifiable in clinical trials in maternal depression and this may be adapted for women with GDM (Holt et al., 2021).

To date no quantitative studies have examined this potential association. In this study, we therefore aimed to examine the relationship between GDM and postpartum mother-infant relationship scores measured at 6 and 15 months postpartum, in an Italian birth cohort.

## 2 Methods

### 2.1 Study design and setting

We used data from the CoNER birth cohort, which recruited women from Bologna, Italy. All women giving birth at the university hospital of Bologna (Sant’Orsola-Malpighi Polyclinic) between June 2004 and December 2005 were considered for inclusion. Individuals were excluded if they were below age 18 years, did not speak or read Italian, or their infant moved to another hospital shortly after birth. The cohort was designed for multipurpose use including research on how environmental and nutritional exposures in early life affect long term outcomes, and the protocol including all its data points and measurements are described elsewhere (Larsen et al., 2013, Porta and Fantini, 2006). A total of 642 women and 645 newborns were recruited at birth, with a participation rate of about 60%. Ethics approval was received from the Comitato etico indipendente dell’Azienda ospedaliero-universitaria di Bologna – Policlinico Sant’Orsola-Malpighi (Submission Number 52/2004/U/Tess, 17.04.2012).

### 2.2 Sample selection

We excluded cases where women had a twin or multiple pregnancy (n = 3), a history of diabetes (type 1 or type 2 diabetes) (n = 6) or unknown history of diabetes (n = 1), resulting in a sample size of 632 women.

### 2.3 Measures: Gestational diabetes mellitus

GDM was self-reported by women at 6 months postpartum via telephone interview. Based on the Italian health guidelines, women were diagnosed with GDM if their fasting plasma glucose level was greater than 5.1 mmol/litre or their 2-hour fasting plasma glucose was greater than 8.5 mmol/litre.

### 2.4 Measures: mother–infant relationship

The mother–infant relationship was assessed via telephone using a purposefully designed tool including 11 items with response options on a Likert scale. Included questions were based on domains including talking, singing, reading books, playing with toys, hugging, playing contact games, walking, bathing and feeding along with breastfeeding habits at both 6 and 15 months. At 6 months, domains related to feeding and playing were also asked and at 15 months, domains related to quarrels and food refusal were also included. An example of a question is “How often do you talk to your baby?”. Due to issues with the reliability of the scale, two items from each scale were removed based on the Cronbach’s alpha, in addition to using the first principal component to maximise reliability. In particular, for the 6-month mother–infant relationship scale, questions assessing feeding and reading books were removed, while for the 15-month mother–infant relationship scale, questions assessing quarrels and food refusal were removed. The Cronbach’s alpha at 6 months was 0.55 and at 15 months was 0.52. For exploratory research, it has been suggested that a value as low as 0.50 is appropriate (Peterson, 1994).

### 2.5 Measures: Covariates

The following variables were collected during pregnancy from medical records: maternal age, education level, occupation status, whether they were breastfeeding at 6 months, mode of delivery, parity, and need for neonatal intensive care unit (NICU) which was later removed due to poor model fit. Depression was measured via telephone interview using the Italian version of the Edinburgh Postnatal Depression Scale (EPDS) (Cox et al., 1987; Carpiniello et al., 1997) at 6 and 15 months postpartum. The EPDS consists of 10 items and requires participants to describe their thoughts and feelings experienced in the past 7-day period using a 4-point Likert scale. The range of scores are 0–30. We recoded depressive scores as a binary variable, with scores above 9 representing caseness for depression (Lanes et al., 2011).

### 2.6 Statistical analysis

We first fitted regression models to linearly estimate the effect of GDM status on mother–infant relationship scores at 6 months and 15 months postpartum in separate analyses. Crude estimates were first calculated followed by adjustment of covariates. We used Monte Carlo Markov Chain (MCMC) Bayesian estimation to fit the models and obtain 95% credible intervals (CrI). MCMC estimation is recommended for robust results when dealing with small and unequal sample sizes (van Ravenzwaaij et al., 2018). Next, we fit a mixed-effects MCMC model to assess the impact of GDM status on mother–infant relationship scores over the 2 time points. Mother–infant relationship scores were nested in individuals, and we included an interaction term to account for change over time. As a sensitivity analysis, we estimated GDM status on the binary depression score at 6 and 15 months using a logistic regression model (supplementary t. 1).

For the linear regression models the burn-in and monitoring chains were set to 500 and 4000 MCMC iterations respectively, and 1000 and 6000 for the mixed-effects model. All models were estimated using weakly informed priors. Model fit was assessed by examining the trajectory plots for chain convergence, effective sample sizes for parameters and the Pareto K estimate. For variables with a high amount of missing data we included missing as a category in the model (not reported in results), while for all else listwise deletion was used. After excluding missing data, we had a final sample size of 576 and 486 for mother-infant relationship scores at 6 months and 15 months, respectively. All data preparation were conducted in R, and analyses were performed using the *Brms* package in R.

## 3 Results

In total, there were 15 women with GDM in the study sample after exclusions. Table 1 presents the background characteristics of the sample by GDM status.

**Table 1:** Background characteristics of the study cohort (n = 632).

	n% missing	No GDM n (%)	GDM n (%)
**Variable**			
**Age (grouped)**	0.6		
18–25		33 (5.4)	0 (0.0)
26–31		183 (30.0)	3 (20.0)
32–37		305 (50.0)	10 (66.7)
38+		89 (14.6)	2 (13.3)

**Depression 6 month**	9.2		
No depression		454 (81.9)	10 (71.4)
Depression		100 (18.1)	4 (28.6)

**Depression 15 months**	19.3		
No depression		454 (92.1)	10 (90.9)
Depression		39 (7.9)	1 (9.1)

**Education**	0.2		
Primary and middle school		110 (18.0)	4 (26.7)
High school		268 (43.9)	8 (53.3)
Master’s degree		232 (38.0)	3 (20.0)

**Employment status**	1.3		
Employed		555 (92.0)	12 (80.0)
Unemployed		13 (2.2)	0 (0.0)
Other		35 (5.8)	3 (20.0)

**Parity**	1.7		
Primiparous		203 (33.8)	7 (46.7)
Multiparous		397 (66.2)	8 (53.3)

**Mode of delivery**	2.9		
Vaginal delivery		477 (80.4)	13 (86.7)
Planned C-section		44 (7.4)	1 (6.7)
Urgent C-section		72 (12.1)	1(6.7)

**Duration of breastfeeding**	9.0		
≥6 months		311 (56.0)	10 (71.4)
<6 months		204 (36.8)	3 (21.4)
Never breastfed		40 (7.2)	1 (7.1)

**Fig. 1:**
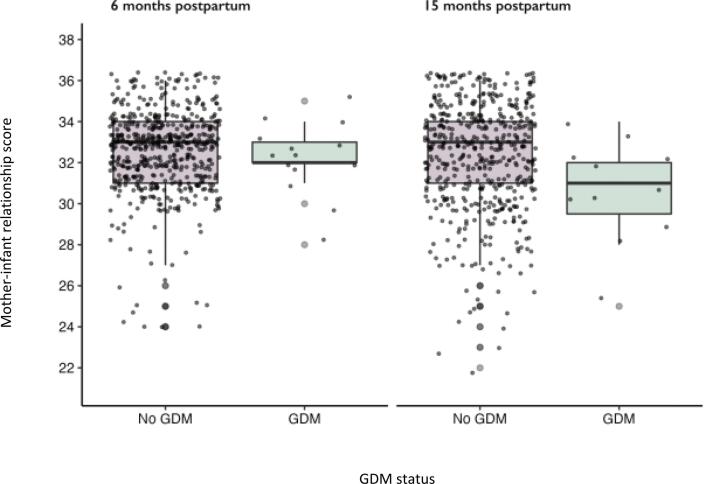
Boxplot of data distribution for mother–infant relationship scores by GDM status at 6 and 15 months postpartum. The following is an explanation of the elements that constitute a boxplot: the median is shown by the line that divides the box into two parts; the range of scores from the lower to upper quartile is marked by the box; whiskers stretch from the lower to the upper adjacent value.

Fig. 1 shows the distribution of data for mother-infant relationship scores by GDM status at 6 and 15 months postpartum. Mean scores [SD] for women without GDM at 6 months and 15 months postpartum were 3.55 [1.99] and 32.28 [2.62], respectively. For women with GDM, mean scores at 6 and 15 months postpartum were 32.21 [1.76] and 30.55 [2.54], respectively (see supplementary T. 2).

We initially estimated through regression analysis the crude effect of GDM status on mother-infant relationship scores at 6 months, which showed no significant association [β − 0.34 95% CrI (−1.41; 0.76)], and at 15 months [β − 1.70 95% CrI (−3.19; −0.24)], which showed GDM predicted significantly lower mother–infant bonding scores compared to women without GDM. After adjustment for all other covariates (Table 2), mother-infant relatinoship scores at 6 months were not signficiantly associated with GDM status [β − 0.27 95% CrI (−1.37; 0.81)], but adjustment for covariates at 15 months strengthed the association between GDM status and lower mother-infant relationship scores [β − 1.75 95% CrI (−3.31; −0.21)]. Never having breastfed predicted lower mother–infant relationship scores at 6 months postpartum, but not at 15 months, compared to women who reported breastfeeding at 6 months [β − 0.93 95% CrI (−1.60; −0.25)]. Having more than one child predicted higher mother–infant relationship scores at both 6 [β 0.47 95% CrI (0.09; 0.84)] and 15 months postpartum [β 0.58 95% CrI (0.04; 1.11)]. The other covariates had no significant impact on mother–infant relationship scores at 6 or 15 months postpartum.

**Table 2:** Mother–infant relationship scores at 6 and 15 months postpartum for GDM and non-GDM groups in the CoNER cohort.

	Mother–infant relationship score at 6 months	Mother–infant relationship score at 15 months
	Beta coefficient (95 %CrI)	Beta coefficient (95 %CrI)
**GDM status**		
*No GDM*	**Ref**	**Ref**
*GDM*	−0.27 (−1.37; 0.81)	**−1.75 (−3.31; −0.21)**

**Depression**		
*No depression*	**Ref**	**Ref**
*Depression*	−0.01 (−0.45; 0.42)	−0.15 (−1.01; 0.68)

**Age group**		
*18–25*	**Ref**	**Ref**
*26–31*	−0.45 (−1.33; 0.40)	0.62 (−0.59; 1.88)
*32–37*	−0.43 (−1.33; 0.41)	0.60 (−0.58; 1.86)
*≥38*	−0.29 (−1.26; 0.62)	0.32 (−1.01; 1.66)

**Birth mode**		
*Vaginal*	**Ref**	**Ref**
*Planned C section*	0.14 (−0.56; 0.82)	0.59 (−0.34; 1.56)
*Urgent C section*	−0.50 (−1.05; 0.03)	0.27 (−0.48; 1.03)

**Breast-fed 6 months**		
*Yes*	**Ref**	**Ref**
*Stopped breastfeeding*	−0.19 (−0.56; 0.17)	−0.37 (−0.84; 0.13)
*Never breastfed*	**−0.93 (−1.60; −0.25)**	0.27 (−0.65; 1.18)

**Parity**		
*Nulliparous*	**Ref**	
*Multiparous*	**0.47 (0.09; 0.84)**	**0.58 (0.04; 1.11)**

**Occupation**		
*Employed*	**Ref**	**Ref**
*Unemployed*	−0.08 (−1.28; 1.12)	0.53 (−1.13; 2.12)
*Other*	0.08 (−0.65; 0.80)	0.73 (−0.24; 1.71)

**Education**		
*Primary/middle school*	**Ref**	**Ref**
*High school*	0.09 (−0.41; 0.58)	−0.05 (−0.77; 0.65)
*Masters*	0.18 (−0.32; 0.69)	−0.12 (−0.86; 0.63)

**Fig. 2:**
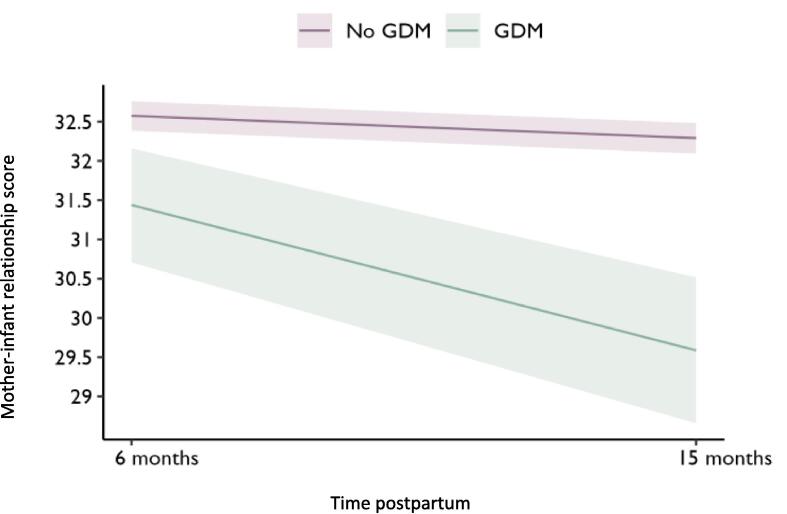
Predicted mother–infant relationship scores from 6 months to 15 months postpartum for women with GDM vs without GDM. Shaded areas correspond to 95% CrIs for predicted mean scores.

Table 3 presents the mixed-effects model. Having GDM showed a significant negative relationship on overall mother–infant relationship scores [β − 1.16 95% CrI (−1.89; −0.41)]. Mother–infant relationship scores were lower at 15 months postpartum compared to 6 months postpartum [β − 0.29 95% CrI (−0.56; −0.02)]. An interaction effect of having GDM and being 15 months postpartum predicted significantly lower mother–infant relationship scores compared to not having GDM and being 6 months postpartum [β − 1.59 95% CrI (−2.41; −0.76)] (Fig. 2).

**Table 3:** Mixed-effects model results of GDM and postpartum time on mother–infant relationship scores.

	Beta coefficient (95 %CrI)
**Intercept**	**32.28 (31.45; 33.12)**

**GDM status**	
*No GDM*	Ref
*GDM*	**−1.16 (−1.89; −0.41)**^1^

**Postpartum time**	
*6 months*	**Ref**
*15 months*	**−0.29 (−0.56; −0.02)**

**Interaction**	
No GDM*6 months	**Ref**
GDM*15 months	**−1.59 (−2.41; −0.76)**

1 Adjusted for age, education, occupation, parity, breastfeeding at 6 months, mode of delivery and depression.

## 4 Discussion

This study aimed to assess the effect of GDM on mother’s perceived relationship with their infant measured at 6 months postpartum and followed up at 15 months. We found that GDM predicted lower mother–infant relationship scores when measured at 15 months postpartum, but not at 6 months. Our findings suggest that the negative impact of GDM on relationship scores may not be apparent within the first-year postpartum, and there may be a delayed response to the experience of GDM. Early physical outcomes of GDM in Western settings are improving for both mother and infant. It may be that women after having undergone a highly medicalised pregnancy to improve physical outcomes, are initially relieved, and only following the initial postpartum period are impacted by the diagnosis at a later time. This interpretation is partially substantiated by a range of qualitative studies in which women have reported considerable fear, anxiety, worry during the pregnancy and relief and later frustration after birth that everything was fine (Craig et al., 2020). Psychoeducation or peer support, beginning in pregnancy and continuing postnatally, should be considered to provide women with better psychological support and preparation for post-GDM experiences.

Interestingly, the sensitivity analysis did not reveal an association between GDM and depressive symptoms, contrasting other studies indicating that GDM increases the risk of depression (Wilson et al., 2020). Other research however, found that although there was a higher prevalence of mental illness amongst women with GDM compared to women without, there was no significant association between GDM and the onset of new depressive or anxiety symptoms both during pregnancy and postpartum (Beka et al., 2018). It may be that the association between GDM and depression is confounded by the presence of depression pre-pregnancy. We also however did not find an association between depressive symptoms and mother–infant relationship scores, contrary to prior studies (Eitenmüller et al., 2022, Tichelman et al., 2019). This may be due to the small sample size in this cohort, or could be due to the stigma associated with postpartum depression that biases the reporting of depressive symptoms. Additionally, as depression was measured postpartum, it is unknown whether pre-pregnancy or during pregnancy depressive symptoms have a greater impact on relationship scores than depressive symptoms measured postpartum. We also do not know in this cohort the lifetime prevalence of depression, which could give more insight into the mechanisms behind depression and an impaired mother–infant relationship, for example, if long-term psychological difficulties or early life adversities are affecting the relationship.

**Strengths and limitations**

This study is to the best of our knowledge the first to quantitatively examine the relationship between mother–infant relationship scores and GDM in the postnatal periods using a birth cohort. The follow-up of women at 2 postnatal time points allowed us to explore how mother–infant relationship scores can change over time. Data were collected from women during pregnancy and postbirth, which may limit the likelihood of recall bias of several key indicators including GDM status. Depression symptomology was also measured using a standardised assessment (EPDS).

Several potential limitations however should be noted. Firstly, the mother–infant relationship questionnaire investigating frequency of bonding activities was purposely designed for the CoNER study and was not validated against gold standard assessment tools. A number of validated tools were considered for inclusion in the questionnaire, such as the Mother–Infant Bonding Scale, the Postpartum Bonding Questionnaire, and the Parent-to-infant Attachment Questionnaire, but were discarded in order to reduce the risk of information bias due to a social desirability effect. Indeed, such scales include straightforward items such as, “I feel distant from my baby”, “I enjoy playing with my baby” or “my baby annoys me”, all statements that may be perceived as sensitive or intrusive, especially in Italy, where the role and duties of motherhood are strongly idealised and perceived as a private affair (Tichelman et al., 2019, Morris, 2018). Secondly, the mother–infant relationship was only assessed postnatally, while it is well established that it begins to develop during pregnancy (Tichelman et al., 2019). Thirdly, differential willingness to participate and loss to follow-up may have introduced bias, and the presence of an interviewer may have led respondents to minimize unpleasant disclosures in order to maximize social acceptability and respectability. Fourthly, due to the overall sample size, there were only a small number of cases of women with GDM. Though we used robust analytical methods to accommodate this, the estimates might not be representative of the wider population and should be interpreted as hypothesis generation for larger cohorts that test the effect of GDM on the mother–infant relationship independent of depressive symptoms with pre-pregnancy depression status included. Lastly, as we used secondary data, there may be residual confounding. There may be unmeasured indicators affecting the mother-infant relatinoship at different postpartum times, for example a subsequent pregnancy may have a greater impact on the relationship at 15 months. The inclusion of factors such as the mothers’ feelings about the pregnancy, stress levels and previous postpartum depression may have provided additional insight or correlates with mother–infant relationships postpartum.

## 5 Conclusion

In conclusion, we found that GDM negatively predicted mother–infant relationship scores at 15 months postpartum, but not at 6 months. Overall mother–infant relationship scores were lower at 15 months than scores reported at 6 months, indicating the impact on the mother–infant relationship extends after the first year after birth. Future research is needed using larger birth cohorts and with validated measures for the mother–infant relationship to further examine the impact of GDM on the mother–infant relationship, and to determine the role of pre-pregnancy depression. Our finding of an association between GDM and impacted mother–infant relationships also points to a need for more research to determine whether this potential impact can be prevented. Interventions during pregnancy for those diagnosed with GDM, such as psychoeducation or counselling during pregnancy and postpartum should be considered. Finally, support for new mothers should extend before the first year after birth, as the negative effect of GDM or indeed other pregnancy complications may be more long term than has previously been considered.

## CRediT authorship contribution statement

**Benton Madeleine:** Writing – review & editing, Project administration, Writing – original draft, Formal analysis, Methodology, Conceptualization. **Davies Megan:** Project administration, Writing – review & editing, Writing – original draft, Formal analysis. **Ismail Khalida:** Writing – review & editing, Project administration, Methodology, Conceptualization. **Lenzi Jacopo:** Writing – review & editing, Project administration, Writing – original draft, Methodology.

## Declaration of Competing Interest

The authors declare that they have no known competing financial interests or personal relationships that could have appeared to influence the work reported in this paper.

## Data Availability

The data that has been used is confidential.
